# Objectively measured physical activity and longitudinal changes in adolescent body fatness: an observational cohort study*

**DOI:** 10.1111/ijpo.12031

**Published:** 2015-04-27

**Authors:** P. J. Collings, K. Wijndaele, K. Corder, K. Westgate, C. L. Ridgway, S. J. Sharp, A. J. Atkin, A. M. Stephen, D. Bamber, I. Goodyer, S. Brage, U. Ekelund

**Affiliations:** ^1^ MRC Epidemiology UnitUniversity of Cambridge School of Clinical MedicineCambridgeUK; ^2^ UKCRC Centre for Diet and Activity Research (CEDAR)MRC Epidemiology UnitUniversity of Cambridge School of Clinical MedicineCambridgeUK; ^3^ MRC Human Nutrition ResearchUniversity of CambridgeCambridgeUK; ^4^ Developmental Lifecourse Research GroupDepartment of PsychiatryUniversity of CambridgeCambridgeUK; ^5^ Department of Sport MedicineNorwegian School of Sports ScienceOsloNorway

**Keywords:** Adiposity, motor activity, prospective study, youth

## Abstract

**Background:**

The data regarding prospective associations between physical activity (PA) and adiposity in youth are inconsistent.

**Objective:**

The objective of this study was to investigate associations between baseline levels of objectively measured PA and changes in adiposity over 2.5 years from mid‐to‐late adolescence.

**Methods:**

This was an observational cohort study in 728 school students (43% boys) from Cambridgeshire, United Kingdom. Fat mass index (FMI, kg m^−2^) was estimated at baseline (mean ± standard deviation age: 15 ± 0.3 years) and follow‐up (17.5 ± 0.3 years) by anthropometry and bioelectrical impedance. Habitual PA was assessed at baseline by ≥3 d combined heart rate and movement sensing. Average daily PA energy expenditure (PAEE) and the time (min d^−1^) spent in light, moderate and vigorous intensity PA (LPA, MPA and VPA, respectively) was estimated. Multilevel models were used to investigate associations between baseline PA and change in FMI (ΔFMI). Adjustment for baseline age, sex, follow‐up duration, area‐level socioeconomic status, season of PA assessment, sedentary time, energy intake and sleep duration was made; baseline FMI was also added in a second model.

**Results:**

FMI increased significantly over follow‐up (0.6 ± 1.2 kg m^−2^, *P* < 0.001). Baseline PAEE and LPA positively predicted ΔFMI in overfat participants (*P* ≤ 0.030), as did VPA in initially normal fat participants (*P* ≤ 0.044). There were further positive associations between PAEE and ΔFMI in normal fat participants, and between MPA and ΔFMI in both fat groups, when adjusted for baseline FMI (*P* ≤ 0.024).

**Conclusions:**

Baseline PAEE and its subcomponents were positively associated with small and unlikely clinically relevant increases in ΔFMI. These counter‐intuitive findings may be explained by behavioural changes during the course of study follow‐up.

**Abbreviations**
FM: fat mass
FMI: fat mass index
LPA: light physical activity
MPA: moderate physical activity
MVPA: moderate‐to‐vigorous physical activity
PAEE: physical activity energy expenditure
SES: socioeconomic status
VPA: vigorous physical activity

## Introduction

Adolescence is a developmental phase that is associated with numerous hormonal, psychological, physical, behavioural and social changes that can contribute to excess body fat acquisition 1. One such change is a decline in physical activity (PA) 2. We have recently shown that 15‐year‐olds engage in modest levels of moderate‐to‐vigorous PA (MVPA) and less than half accumulate the recommended 60 min of MVPA per day (MVPA d^−1^) 3.

Cross‐sectional studies have consistently reported marked inverse associations between PA and adiposity, implying that low levels of PA may predispose to high youth body fatness 4, 5. They have further indicated that higher intensity PA may be more strongly associated with adiposity than activities of low‐to‐moderate intensity 6, 7, 8. However, there is no convincing evidence for a prospective association between these constructs 9, 10, and a recent systematic review concluded that objectively measured PA may not be a key determinant of adiposity gain 11. It remains problematic, nonetheless, that the available literature is hindered by methodological limitations. These include imprecision in the measurements of exposure and outcome, short durations of follow‐up and inadequate adjustment for potential confounders.

The objective of this study was to investigate associations between PA and adiposity over 2.5 years from mid‐to‐late adolescence. We used combined heart rate and movement monitoring to estimate PA energy expenditure (PAEE) and its sub‐components in 728 UK adolescents, and adjusted our analyses for a range of potential confounding factors.

## Methods and procedures

### Study population and project outline

The ROOTS study is a community‐based prospective cohort study set in the East of England 12. Twenty‐seven secondary schools located within the city of Cambridge and adjacent villages were approached to participate in ROOTS. Eighteen schools agreed to do so, from which 3762 eligible adolescents and their families were invited to the study. Recruitment peaked at 1238, and 1203 adolescents attended for testing (32% of those approached).

Data collection began at Wave 0 (commencing April 2005 and continuing until January 2007) with all participants and one parent (usually the mother) visiting schools for collection of demographic data by self‐report and interview. Adolescent body composition was also measured. About 6 months after Wave 0 (between December 2005 and July 2007), all participants were invited for another assessment of body composition and an initial measurement of habitual PA (Wave 1, interchangeably referred to as baseline). The invitation was accepted by 930 adolescents (43% boys; 75% of Wave 0 participants). Approximately 2.5 years after Wave 1, 844 participants (44% boys; 68% of Wave 0 participants) were followed for third and final measurements of body composition at Wave 2 (interchangeably referred to as follow‐up). Wave 2 ran from January 2008 and finished in March 2010, and also included another assessment of PA in a random subgroup of 212 adolescents (47% boys; 17% of Wave 0 participants). All data collections were conducted during school terms.

The ROOTS project received ethical approval from the Cambridge Local Research Ethics Committee (reference number 03/302) and was conducted in agreement with Declaration of Helsinki guidance. All participants and their parent(s) provided written informed assent/consent and were free to decline all or part of the study without penalty.

### Assessment of body composition

Detailed physical measures were made by trained personnel at all waves. Height was measured to the nearest 0.1 cm (Leicester Height Metre; Invicta Plastics, Leicester, UK) while barefoot and in light clothing and weight to the nearest 0.1 kg (Tanita TBF‐300 MA, Tanita, Tokyo, Japan) using standard procedures. Body mass index (BMI, kg m^−2^) was calculated. Body tissue impedance (Ω) was measured by bioelectrical impedance analysis (BIA) weighing scales (Tanita TBF‐300 MA). With these data, estimates of fat mass (FM, kg) were derived 3, 13 and converted to the FM index (FMI, kg m^−2^) by division of the square of height in metres. Unlike percentage of body fat, FMI permits independent evaluation of adiposity relative to body size 14. Internally derived age‐ and sex‐specific FMI cut‐offs were used to categorize participants as normal (<85th percentile) or overfat (≥85^th^ percentile) at baseline.

### Assessment of PA

A detailed description of the activity measurements is available elsewhere 3. In brief, PA was measured at baseline and follow‐up (in a subsample) by combined heart rate and movement sensing (Actiheart, CamNtech, Ltd, Cambridge, UK) following individual calibration of heart rate by a ramped sub‐maximal step‐test performed at baseline 15. The water‐resistant monitor (attached to the chest by two electrocardiogram electrodes) recorded data every 30 s and was worn without interruption (including sleep) for 4 consecutive days inclusive of a weekend.

Metabolic equivalents of thermogenesis (MET; 1 MET defined as 3.5 mL O_2_ kg^−1^ min^−1^) were used to estimate the daily time (min d^−1^) spent in light PA (LPA, >1.5–4 METs), moderate PA (MPA, >4–7 METs) and vigorous PA (VPA, >7 METs). Total sedentary time (≤1.5 METs inclusive of sleep) was also derived, with self‐reported bed times and visual inspection of plotted objective data permitting disaggregation of this construct into objectively measured total sleep duration (≤1.5 METs inside of sleep time) and sedentary time (≤1.5 METs outside of sleep time) 3. In analyses, both sleep duration and sedentary time were regarded as potentially important confounding factors. Valid PA data were defined as free‐living periods that contained ≥32 h of weekday and ≥16 h of weekend data, with an additional proviso that these hours should be equally distributed across all parts of days (≥12 h recorded at morning [03:00–09:00 h], noon [09:00–15:00 h], afternoon [15:00–21:00 h] and midnight [21:00–03:00 h] across the whole observation period).

### Other variables

A demographic questionnaire, completed at Wave 0, collected information regarding participant sex, ethnicity (dichotomized as White or other) and postcode. The latter was used to create a three‐category (low, middle or high) area‐level socioeconomic status (SES) variable 3. Age at parturition and birth weight were parentally reported as part of an obstetrics history questionnaire; both were treated as continuous variables. A composite pubertal status marker (pubertal vs. non‐pubertal) was also determined at Wave 0 using a combination of methods including menarcheal status, self‐reported tanner staging and salivary testosterone levels in boys, as described elsewhere 3.

At baseline, diet diaries were completed by adolescents, in which they recorded all food and drink consumed over the same 4 d that PA was measured. Diaries were coded and analysed to provide information on average daily energy intake (MJ d^−1^) using ‘Diet In Nutrients Out’ (DINO), an in‐house dietary assessment system based on McCance and Widdowson's ‘The Composition of Foods’ 16.

The Mood and Feelings Questionnaire (MFQ), a 33‐item scale designed to elicit information about recent depressive symptoms in children and adolescents 17 was completed by participants at Waves 0 and 2. This information was retained in continuous form and included in analyses as a time‐varying factor. At 18 months post Wave 0 (a stage in the ROOTS study primarily used to collect psychosocial measures that is not otherwise used in this investigation) height and weight were measured in 428 mothers whose children had valid PA data at baseline. These data were used to compute contemporary maternal BMI, which because of the extent of missing data, was considered as part of sensitivity analyses only.

### Statistical analyses

#### Descriptive statistics

The analysis was restricted to participants with valid baseline PA data, the demographic and PA characteristics of which were summarized using means, standard deviations, medians, interquartile ranges (IQRs) and percentages. Correlations were calculated to quantify the association between the baseline PA components and their change over time. A mixed‐model analysis of variance (anova) was used to investigate ΔFMI. To examine differences in Wave 0 adiposity among participants with and without valid PA data the Wilcoxon rank–sum test was used. Finally, for participants with valid PA data but missing covariate or follow‐up adiposity data, the assumption that the data were missing completely at random (MCAR) was assessed using Little's MCAR chi‐squared test 18.

#### Main analysis

Associations between baseline PA and ΔFMI were estimated using multilevel models 19. All models had FMI as the dependent variable, a single independent predictor variable (PAEE, LPA, MPA or VPA), and two levels: (i) phase of data collection (baseline or follow‐up) and (ii) participant. Models were specified with random intercepts and slopes at the participant level. School was initially included as a third level, but was dropped as it explained no variation in the outcome; accounting for school clustering did not influence the descriptive analyses either. The multilevel model is described in full as Appendix S1.

Model 1 included basic demographic factors (baseline age, sex, area‐level SES, follow‐up duration), season of baseline assessment, sedentary time and sleep duration. All other potential confounding variables (ethnicity, pubertal status, energy intake, depressive symptoms, birth weight and maternal age at parturition) had missing values. To preserve sample size, these variables were added one at a time to Model 1 and were only retained if they changed the magnitude of association between PA and ΔFMI by >10% 20. For Model 2, baseline FMI was added to Model 1. Based on previous work 21, 22, 23, 24, interactions between PA and sex, as well as between PA and baseline body fat group, were investigated. As FMI was skewed and natural log‐transformed prior to analyses, the data have been back‐transformed by exponentiation, to represent the expected percentage ΔFMI per unit difference in independent variables.

Analyses were performed using Stata version 13.1 (StataCorp, College Station, TX, USA) except for Little's MCAR chi‐squared test which was performed in SPSS version 11.0 (IBM, Chicago, IL, USA).

## Results

Valid PA data were available for 736 participants, but energy intake consistently changed the magnitude of association between PA and ΔFMI by >10%. The final sample was therefore restricted to 728 participants (43% boys) with complete data for energy intake. Included participants were leaner compared with the 475 adolescents who were either measured at Wave 0 but who did not undergo PA testing (*n* = 273), did not provide sufficient valid activity data (*n* = 194), or had missing data for energy intake (*n* = 8) (FMI: median 4.2 [IQR 3.0] vs. 4.5 [3.3] kg m^−2^, *P* = 0.042).

Table 1 contains participant characteristics stratified by baseline body fat status. Participants were 15 years of age at baseline and were followed for approximately 2.5 years. Ethnic minority representation was low (95% of participants were White) and the majority of participants (61%) lived in high SES areas. Compared with participants with normal body composition, overfat participants reported lower energy intake and had mothers with higher BMI (*P* ≤ 0.01). With the exception of area‐level SES, sedentary time, energy intake and sleep duration, there were some missing values for all other potential confounders. In addition, FMI was missing for 183 participants at follow‐up (75% retention of contributing participants), but Little's test provided no evidence against the assumption that all covariate and outcome data were MCAR (*P* = 0.28).

**Table 1:** Characteristics of participants stratified by baseline body fat group*

	All (*n* = 728)	Normal (*n* = 619)	Overfat (*n* = 109)
Age (years)			
Wave 0	14.5 ± 0.3^†^	14.5 ± 0.3	14.5 ± 0.3
Wave 1 (baseline)	15.0 ± 0.3	15.0 ± 0.3	15.0 ± 0.4
Wave 2 (follow‐up)	17.5 ± 0.3	17.5 ± 0.3	17.5 ± 0.3
Follow‐up duration (years)	2.4 ± 0.2	2.4 ± 0.2	2.5 ± 0.3**
Sex (%)^‡^			
Boys	43.0	42.3	46.8
Girls	57.0	57.7	53.2
Ethnicity (%)			
White	94.7	94.9	93.4
Other	5.3	5.1	6.6
Area‐level SES (%)			
Low	15.0	15.0	14.7
Middle	23.6	23.4	24.8
High	61.4	61.6	60.6
Pubertal status (%)			
Pre‐pubertal	9.4	9.6	8.4
Pubertal	90.6	90.4	91.6
Maternal age at parturition (years)	29.0 ± 5.0	29.1 ± 5.1	28.7 ± 4.6
Birth weight (kg)	3.38 ± 0.56	3.37 ± 0.57	3.44 ± 0.49
Energy intake (MJ d^−1^)	7.63 ± 2.12	7.74 ± 2.16	7.03 ± 1.75^††^
Depressive symptoms			
Wave 0	12 (13)^§^	12 (13)	14 (11)
Follow‐up	12 (12)	12 (12)	12 (11)
Sleep duration (h per night)	8.1 ± 0.8	8.1 ± 0.7	8.0 ± 0.8
Sedentary time (min d^−1^)	367.7 ± 114.0	365.8 ± 113.0	380.0 ± 119.0
Maternal BMI (kg m^−2^)^¶^	24.4 (5.8)	24.0 (5.5)	27.3 (7.7)^‡‡^

*Body fat groups based on age‐ and sex‐specific fat mass index cut‐offs with ≥85th percentile denoting overfat participants (<15.0 years: boys ≥4.49 kg m^−2^, girls ≥7.48 kg m^−2^; >15.0 years: boys ≥4.86 kg m^−2^, girls ≥7.98 kg m^−2^); there were missing data for Wave 2 age and follow‐up duration (*n* = 183 for both), ethnicity (*n* = 13), pubertal status (*n* = 15), maternal age at parturition (*n* = 18), birth weight (*n* = 29), depressive symptoms (*n* = 28 at Wave 0; *n* = 103 at Wave 2) and maternal BMI (*n* = 301); Little's χ^2^ test provided no evidence against the assumption that these data were missing completely at random (*P* = 0.28). ^†^Mean ± SD (for all such values), body fat group comparisons made by analysis of variance, there were no differences in results when controlling for gender. ^‡^Body fat group comparisons made by χ^2^ tests (for all categorical variables). ^§^Median and IQR in parentheses (for all such values), body fat group comparisons made by Wilcoxon rank–sum test. ^¶^Included only in sensitivity analyses because of substantial missing data. **Significant difference between body fat groups, *P* < 0.05; ^††^Significant difference between body fat groups, *P* < 0.01; ^‡‡^Significant difference between body fat groups, *P* < 0.001. BMI, body mass index; IQR, interquartile range; SD, standard deviation; SES, socioeconomic status.

**Figure 1:**
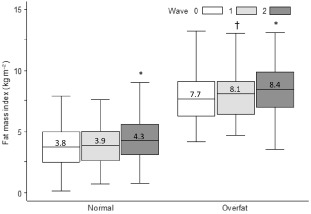
Median (IQR) FMI at Waves 0, 1 (baseline) and 2 (follow‐up) stratified by baseline body fat group. Values inside the boxplot represent medians and all values outside 1.5× IQR are excluded; data analysed by mixed‐model analysis of variance; Regression of FMI against height showed that variables were not associated (*r* ^2^ = 0.23 & *P* = 0.78 in a sex‐adjusted model); †Significant difference from Wave 0, *P* = 0.028; *Significant difference from Waves 0 and 1, *P* < 0.001; overall ΔFMI from Wave 0 to 2 was not different between the body fat groups, *P* = 0.19; FMI, fat mass index; IQR, interquartile range.

Figure 1 shows median (IQR) FMI levels at all waves stratified by baseline body fat status. There was no significant change in FMI over the short duration (0.5 year) between Waves 0 and 1 in the normal fat group (*P* = 0.38), but all other gains in FMI between successive waves were statistically significant (*P* ≤ 0.028). The magnitude of overall ΔFMI was indifferent between baseline body fat groups (*P* = 0.19 for body fat group × wave interaction). There was also no difference in mean ΔFMI between genders (*P* = 0.19).

**Figure 2:**
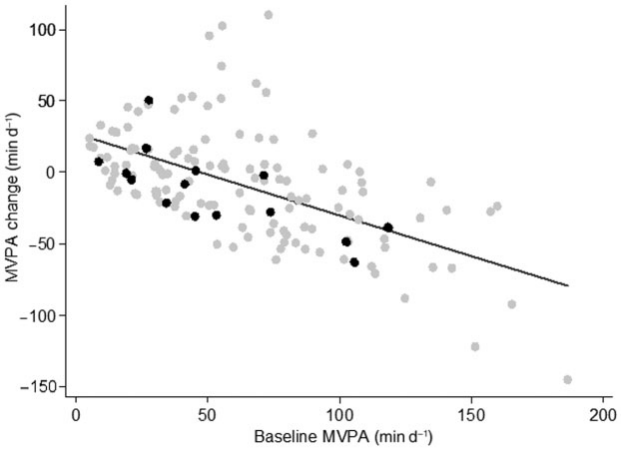
Scatterplot with trend line of the relationship between change in MVPA from baseline to follow‐up with the baseline level of MVPA. MVPA, moderate‐to‐vigorous physical activity (>4 metabolic equivalents of thermogenesis); black scatter points indicate overfat participants; *n* = 144.

Table 2 shows the PA levels of participants at baseline along with details regarding changes in PA over follow‐up (the latter was available for only 144 individuals with valid repeated PA data). Participants with body fat levels in the overfat range had lower levels of PAEE, MPA and VPA at baseline compared with normal fat participants (*P* ≤ 0.05); the difference was most marked for VPA. Consistently, in the subsample of participants with data for change in PA, the rate of change was negatively correlated with baseline levels. In overfat participants, the decline in MPA was most strongly correlated with its baseline level, whereas in the normal fat group there was a tendency for correlations to be higher with increasing PA intensity. Figure 2 plots the change in MVPA against the baseline MVPA level.

**Table 2:** PA profile of participants stratified by baseline body fat group*

	*n*	All	Normal	Overfat
Baseline PAEE (kJ kg^−1^ d^−1^)	728	74.2 ± 23.8^†^	75.0 ± 24.0	69.1 ± 22.1^¶^
ΔPAEE over follow‐up (kJ kg^−1^ d^−1^)	144	−2.3 ± 20.7	−2.0 ± 21.3	−4.4 ± 14.8
*r* between baseline and Δ	144	−0.40^‡^	−0.41	−0.32
Baseline LPA (min d^−1^)	728	518.5 ± 106.5	517.7 ± 106.5	523.1 ± 106.8
ΔLPA over follow‐up (min d^−1^)	144	1.8 ± 102.2	3.2 ± 100.4	−9.6 ± 120.3
*r* between baseline and Δ	144	−0.27	−0.32	0.22
Baseline MPA (min d^−1^)	728	48.0 (41.1)^§^	49.1 (40.8)	41.4 (40.5)^¶^
ΔMPA over follow‐up (min d^−1^)	144	−5.3 ± 30.2	−5.0 ± 31.1	−7.8 ± 21.8
*r* between baseline and Δ	144	−0.53	−0.52	−0.65
Baseline VPA (min d^−1^)	728	7.1 (19.7)	7.7 (21.1)	3.9 (13.7)**
ΔVPA over follow‐up (min d^−1^)	144	−3.5 ± 14.6	−3.4 ± 15.0	−4.4 ± 11.0
*r* between baseline and Δ	144	−0.56	−0.56	−0.53

*Body fat groups based on age‐ and sex‐specific fat mass index cut‐offs with ≥85th percentile denoting overfat participants; Baseline PA levels based on 728 participants (normal: *n* = 619; overfat: *n* = 109); changes in PA over follow‐up and correlations with the baseline level are based on 144 participants only (normal: *n* = 129; overfat: *n* = 15). ^†^Mean ± SD (for all such values), body fat group comparisons for baseline activity made by analysis of variance, there were no differences in results when controlling for gender. ^‡^Spearman correlation coefficients between baseline PA and its change from baseline to follow‐up (for all such values). ^§^Median and IQR in parentheses (for all such values), body fat group comparisons for baseline activity made by Wilcoxon's rank–sum test. ^¶^Significant difference between body fat groups, *P* < 0.05. **Significant difference between body fat groups, *P* < 0.001. IQR, interquartile range; LPA, light physical activity; MPA, moderate physical activity; PA, physical activity; PAEE, physical activity energy expenditure; SD, standard deviation; VPA, vigorous physical activity.

Table 3 displays the results from multilevel models examining PA variables as predictors of ΔFMI. The associations did not differ between the sexes (*P* ≥ 0.43 consistently for sex interactions) so models were fit in boys and girls combined and adjusted for sex. There were, however, consistent statistically significant interactions between baseline FMI and PA (*P* < 0.001 for all bar one *P*‐value; remaining *P* = 0.082), so results are shown stratified by baseline body fat group. Results from Model 1 showed that VPA was positively associated with ΔFMI in the normal fat group (*P* = 0.044). In overfat participants, PAEE and LPA were both positively associated with ΔFMI (*P* ≤ 0.030). All confidence intervals from Models 1 and 2 overlapped, but point estimates were generally larger when adjusted for baseline FMI in Model 2. This translated to a significant positive association between PAEE and ΔFMI in the normal fat group (*P* = 0.017), and significant associations between MPA and ΔFMI in both fat level groups (*P* ≤ 0.024), when adjusted for baseline FMI. All results were essentially unchanged when maternal BMI was introduced as a covariate and when sedentary time was omitted from analyses amidst concerns for multicollinearity.

**Table 3:** Associations between baseline PA and changes in adiposity (stratified by baseline body fat group)*

	Annual percentage change (95% CI) in ΔFMI (kg m^−2^)			
	Normal (*n* = 619)	*P*‐value	Overfat (*n* = 109)	*P*‐value
Baseline PAEE (kJ kg^−1^ d^−1^)				
Model 1	0.26 (−0.089 to 0.60)	0.15	1.55 (0.40 to 2.70)	0.008
Model 2	0.43 (0.076 to 0.79)	0.017	1.73 (0.52 to 2.95)	0.005
Baseline LPA (min d^−1^)				
Model 1	−0.023 (−0.095 to 0.048)	0.52	0.21 (0.021 to 0.40)	0.030
Model 2	−0.0012 (−0.069 to 0.067)	0.97	0.21 (0.021 to 0.41)	0.030
Baseline MPA (min d^−1^)				
Model 1	0.17 (−0.096 to 0.45)	0.21	0.82 (−0.039 to 1.69)	0.061
Model 2	0.36 (0.048 to 0.67)	0.024	1.83 (0.55 to 3.12)	0.005
Baseline VPA (min d^−1^)				
Model 1	0.46 (0.012 to 0.92)	0.044	0.78 (−1.15 to 2.74)	0.43
Model 2	0.81 (0.20 to 1.42)	0.009	2.13 (−0.65 to 4.99)	0.14

*Body fat groups based on age‐ and sex‐specific fat mass index cut‐offs with ≥85th percentile denoting overfat participants; separate multilevel models with random participant intercept and slopes were constructed for each PA variable; values represent the annual percentage change in adiposity per unit change in activity (calculated by the formula: {[exp(*β* × scaling factor) − 1] × 100} ); PAEE expressed per 10 kJ kg^−1^ d^−1^, which is equivalent to approximately 45 min of walking at 4 METs; light, moderate and vigorous intensity PA expressed per 10 min d^−1^; Model 1 includes age at baseline, sex, follow‐up duration, area‐level SES, season of baseline assessment, sedentary time, sleep duration and energy intake; Model 2 is specified as per Model 1, but further adjusted for baseline FMI. CI, confidence interval; FMI, fat mass index; LPA, light physical activity; MET, metabolic equivalent of thermogenesis; MPA, moderate physical activity; PA, physical activity; PAEE, physical activity energy expenditure; SES, socioeconomic status; VPA, vigorous physical activity.

## Discussion

### Summary of results

We found positive associations between baseline PAEE, LPA, MPA and VPA with ΔFMI. Point estimates were generally larger in participants who were overfat. This somewhat resembles the observation of Stevens *et al*. 22, who reported a positive association between accelerometer counts and increased FM in overweight children. However, we also found positive associations in normal fat children, where there was a pattern of stronger associations with increasing PA intensity. Importantly, the magnitude of associations were all weak and are unlikely clinically relevant. The largest significant beta coefficient was equal to a modest 1.83% or 0.002 kg m^−2^ annual increase in the ΔFMI in the initially overfat group, per 10 min d^−1^ of baseline MPA.

### Comparison with previous research

We are aware of another 16 prospective studies that have examined associations between baseline objectively measured PA (assessed by DLW or accelerometry) with longitudinal changes in adiposity (frequently assessed by DXA or BIA) in childhood or youth 21, 22, 23, 24, 25, 26, 27, 28, 29, 30, 31, 32, 33, 34, 35, 36. Four studies have reported qualitatively different beta‐coefficients according to subgroups (i.e. boys vs. girls; normal vs. overweight) or different stages of follow‐up 22, 23, 24, 35. For instance, one of these studies reported a significant negative association in girls only (combined with a positive non‐significant beta in boys) 23. Elsewhere, a significant positive relation was found in girls alongside a significant negative association in boys 24. As already alluded, Stevens *et al*. 22 found significant negative associations in normal weight but positive associations in overweight participants. Another study has reported a beta coefficient of exactly zero 26. Conversely, five studies have consistently reported inverse beta‐coefficients between baseline PA and adiposity change 21, 31, 32, 33, 36. One of the five studies reported non‐significant results 32, two studies reported statistically significant results in both sexes 31, 33, the fourth reported significance in boys only 21, and the fifth found a protective effect of PA on obesity risk in White, but not Black girls 36. In contrast to these investigations, and in agreement with the current work, a handful of studies have reported counter‐intuitive positive beta‐coefficients between baseline PA and changes in adiposity. Specifically, four studies 28, 29, 30, 34 have reported positive albeit small and non‐significant beta‐coefficients for the association between variables, while two studies have reported statistically significant positive effects 25, 27.

It seems that the nature of the prospective relationship between PA and fatness in childhood may be more complex than first appreciated. Alternatively, changing PA behaviours over follow‐up may offer a plausible explanation for the observed paradoxical relationships 37. A lower activity level with advancing age is an often replicated finding, and a recent pooled analysis of mainly self‐reported activity concluded that the annual decline in PA across adolescence is equal to −7.0% (95% confidence interval: −8.8 to −5.2) 2. It has also been shown that the magnitude of decrease in PA is associated with the baseline level 2, 38. In other words, adolescents who are more active at baseline experience larger decreases in activity over follow‐up, compared with adolescents who initially have lower levels. This may be unsurprising because of the marked decline in PA that occurs through adolescence combined with flooring effects (participants beginning at lower levels have less opportunity for decline). Accordingly, we too observed in our subgroup who had repeated measurements of valid PA data (*n* = 144) an inverse relationship between baseline and change in activity. Assuming that the correlations between baseline PA and its change from baseline to follow‐up in the subsample (reported in Table 2) are representative of all participants involved in our main analysis, this may provide some explanation for our unexpected results. Potentially, participants with higher baseline PA levels may have acquired more adiposity over time not because of their initial high activity level, but because they experienced larger declines in PA over follow‐up compared with their initially less active peers.

With this in mind, future investigations may need to account for follow‐up PA or changes in activity over follow‐up if they are to yield valid results when investigating baseline PA and its association with change in adiposity. This is particularly true if the PA level is not stable. However, we are aware that regression to the mean may have contributed to the strength of our reported correlations between baseline PA and its change from baseline to follow‐up. Regression to the mean is a phenomenon whereby extreme measurements at one time‐point are followed by subsequent measurements that tend to be closer to the average. Figure 2 illustrates this concept, by showing that participants with higher baseline PA levels not only had the greatest activity decline over follow‐up, but also that there was a tendency for some participants with initially low PA levels to increase in activity over time. It is further conceivable that changes in other lifestyle behaviours may explain our results, such as changes in energy intake over follow‐up. Unfortunately, no data were available in the current study to elucidate this.

### Strengths and limitations

Over 700 adolescents provided valid PA data, and the vast majority (75%) were retained over 2.5 years of follow‐up. We measured body composition using a pooled estimation method which has been shown to be more accurate than single estimations 13, but there will still be error and potential misclassification between FM and fat‐free mass. Our combined heart rate and movement method appears to be more valid for predicting PAEE 39 and low‐to‐moderate intensity PA 40 compared with motion sensors that are based on acceleration only. A limitation, nonetheless, is the small fraction of follow‐up participants undergoing repeated PA assessment, as changes in PA over time may have exerted a prominent influence on our associations. Although participant retention was satisfactory, the recruitment rate was relatively low, with only one‐third of all invited school students willing to participate in ROOTS. This level of acceptance places the study at some risk of non‐response bias. Indeed, participants with valid baseline PA data were leaner at Wave 0 compared with all remaining ROOTS participants that did not contribute to this specific analysis. We have also previously discussed that ROOTS adolescents are leaner compared with national and regional age‐matched equivalents 3. Nonetheless, it is reassuring that our participants were representative of Cambridgeshire in terms of ethnicity (predominantly White) and SES (middle‐to‐high).

## Conclusion

We observed counter‐intuitive positive associations between baseline PA volume and intensity with changes in adiposity from middle‐to‐late adolescence. These associations are potentially attributable to changes in PA contingent upon the baseline level. Future studies may need to measure body fatness and PA simultaneously at follow‐up, or at multiple time‐points, to account for changes in behaviour over the course of an investigation.

## Conflict of interest statement

No conflict of interest was declared.

## Author contributions

IG designed the research; KC, CLR and DB helped to coordinate and conduct the research; AMS compiled the diet questionnaire; KWe assisted PJC in processing activity data; PJC performed all additional statistical analyses, conducted the literature review and drafted the paper; SJS assisted statistical analyses; UE and SB designed the research, conceived the study and helped draft the paper. All authors, including KWi and AJA, helped to interpret the findings and read, revised and approved the final paper.

## Supporting information


**Appendix S1.** log(FMI_ij_) = β_0_ + u_i0_ + β_1_ × age_i0_ + β_2_ × time_ij_ + β_3_ × PA_i0_ + (γ + u_1i_) × (time_ij_*PA_i0_) + covariates + ε_ij_.
