# Supplementary material for: Objectively measured physical activity and longitudinal changes in adolescent body fatness: an observational cohort study
Source: Pediatr Obes. 2015 Apr 27;11(2):107–14. doi: 10.1111/ijpo.12031 (PMC4780592; doi:10.1111/ijpo.12031)
Supplement: Supplementary file 1 — Appendix S1. log(FMIij) = β0 + ui0 + β1 × agei0 + β2 × timeij + β3 × PAi0 + (γ + u1i) × (timeij*PAi0) + covariates + εij. [file IJPO-11-107-s001.doc]

**log(FMI_ij_) = β_0_ + u_i0_ + β_1_ x age_i0_ + β_2_ x time_ij_ + β_3_ x PA_i0_ + (γ + u_1i_) x (time_ij_*PA_i0_) + covariates + ε_ij_**

Participant level random effects:

u_0i_ ~ N (0, σ_0_^2^)

u_1i_ ~ N (0 , σ_1_^2^)

Wave level residuals:

ε_ij_ ~ N (0, σ_ε_^2^)

i=individual (i=1 to 728)

j=measurement occasion (j=0,1,2)

time_ij_ = years since baseline of measurement occasion j for individual i. time_i0_ = 0.

γ can be interpreted as the average effect of baseline PA on the change in log(FMI) over time.
